# Hazardous waste management and one health approach

**DOI:** 10.3389/fpubh.2025.1684269

**Published:** 2025-10-07

**Authors:** Pinelopi Petropoulou

**Affiliations:** Department of Nursing, University of West Attica, Aigaleo, Greece

**Keywords:** hazardous waste, medical waste, one health, public health, cancer

## Introduction

Solid waste comprises diverse categories, including municipal, industrial, construction and demolition, agricultural and livestock, biomedical, and hazardous waste, each with distinct sources and potential health and environmental impacts. Ineffective management of these wastes can lead to soil, water, and air pollution, facilitate the spread of pathogens, and contribute to antimicrobial resistance, while also affecting food safety and ecosystem integrity. Municipal and industrial wastes introduce chemical and biological hazards, construction and demolition materials increase landfill pressures and environmental degradation, and agricultural and healthcare wastes pose risks of pathogen dissemination and chemical contamination. Hazardous and electronic wastes further threaten biodiversity and long-term ecological balance. Taken together, these issues underscore the interconnected nature of human, animal, and environmental health and highlight the critical importance of implementing waste management strategies within the One Health framework (1).

Landfilling is one of the most widely used waste management methods across all countries, regardless of their level of development. The main types of landfills include (a) municipal solid waste landfills, (b) industrial waste landfills, and (c) hazardous waste landfills. In most cases, these facilities are designed and regulated to ensure that waste disposal complies with specific quality and quantity standards. However, in many developing countries, illegal and uncontrolled “open dumps” remain a common issue, leading to the release of gases such as CO_2_, H2S, CH_4_, and NOx into the atmosphere. These emissions have been linked to respiratory diseases and certain forms of cancer, posing a higher risk to children living in nearby areas. To mitigate these risks, it is crucial to implement advanced waste management technologies, enforce stricter landfill regulations, and establish greater minimum distance requirements between landfills and residential areas. Pollutants can be categorized into inorganic, organic, and biological types. Organic pollutants include domestic, agricultural, and industrial wastes that harm the health and survival of both animals and human populations. Inorganic pollutants primarily consist of potentially toxic elements (PTEs), such as mercury (Hg), lead (Pb), and cadmium (Cd). These substances tend to bioaccumulate within trophic chains, thereby posing significant risks to terrestrial and aquatic organisms. Additionally, biological pollutants of anthropogenic origin are present in the environment, with principal representatives including viruses, bacteria, and various pathogenic microorganisms (1, 2).

“Ineffective waste management” refers to practices where solid waste is collected, treated, or disposed of in ways that fail to prevent adverse impacts on public health, ecosystems, and the broader environment. From a One Health perspective, ineffective waste management creates pathways for the transmission of pathogens, toxic substances, and pollutants across species and ecosystems. For instance, uncontrolled landfilling and open dumping provide breeding grounds for vectors such as flies, rodents, and mosquitoes, which can spread zoonotic diseases to humans and domestic animals (3).

The One Health approach, which acknowledges the interconnectedness of human, animal, and environmental health, provides a holistic solution to these challenges. In the context of solid waste management, the One Health approach highlights how improper handling of municipal, industrial, agricultural, or biomedical waste can simultaneously affect ecosystems, animals, and human populations. For example, unmanaged landfills or open dumping can facilitate the spread of zoonotic pathogens via vectors, contaminate water and soil with toxic substances, and increase antimicrobial resistance—all of which demonstrate interconnected health risks across species and environments (4).

Applying a One Health perspective to waste management therefore entails not only improving technical disposal methods but also integrating policies, surveillance, and practices that concurrently protect human health, animal welfare, and ecosystem integrity. This holistic approach has been increasingly promoted in global health strategies as essential for sustainable environmental governance (5).

By promoting interdisciplinary collaboration, community engagement, policy enhancement, institutional capacity building, and public-private partnerships, this approach plays a key role in ensuring environmental sustainability. The successful implementation of One Health strategies requires coordinated efforts from governments, local communities, private sector stakeholders, and international organizations to create a cleaner and healthier environment (6).

Although increasing attention has been given to the health and environmental impacts of solid waste, significant research gaps persist. This studt comes to emphasize that limited evidence exists on the integrated application of the One Health framework, the long-term and synergistic effects of pollutants such as PAHs, heavy metals, and e-waste, and the effectiveness of community-based interventions and digital innovations in reducing risks.

## Aim-methodology

The purpose of this study is to highlight the public health risk from the incorrect management of hazardous waste by the community and the state, while suggesting ways to effectively manage it.

**Figure 1:**
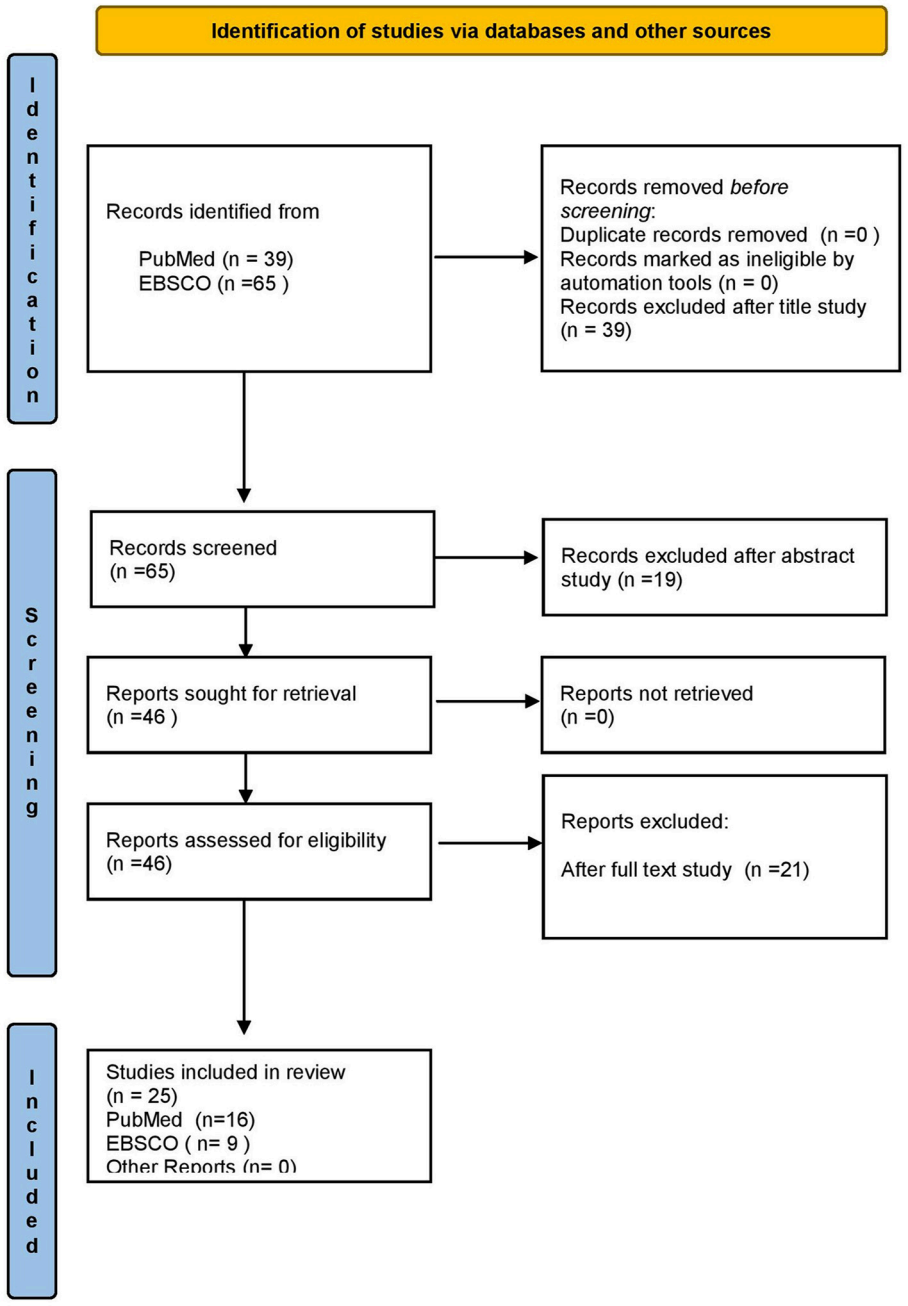
Flow chart of the included studies.

A narrative review was conducted in the PubMed and EBSCO databases using the Boolean search string “Hazardous Waste” AND “One Health Approach”. A total of 39 results were identified in the PubMed database searching for all types of studies from 2019 to 2024 that provided the full research text via open access of which 23 were rejected after the full-text study; thus, 16 articles were included in the present analysis. Also, after searching the EBSCO database with the same key words and the same study selection criteria no duplicate studies were found. Due to the research gap for the specific topic we examined in this particular database, there were few references, so the research was chosen for all type of studies over time and found 65 studies were found that investigated this topic and 9 were included in this study.A total of 25 articles are included in this study according to the inclusion criteria (Figure 1).

### Inclusion criteria for articles in the study

PubMed: all types of studies conducted in the last 5 years, with free full text and abstract available. EBSCO: all types of studies over time, with free full text and abstract available, published in respected academic journals.

All papers had to examine the connection between hazardous waste management and public health so as to be included in this study.

Study limitations: all articles are written in English or Greek. The present study is an opinion piece/narrative review; therefore, the flow diagram is provided to facilitate the presentation and the selection of the material.

## Results

**Figure 2:**
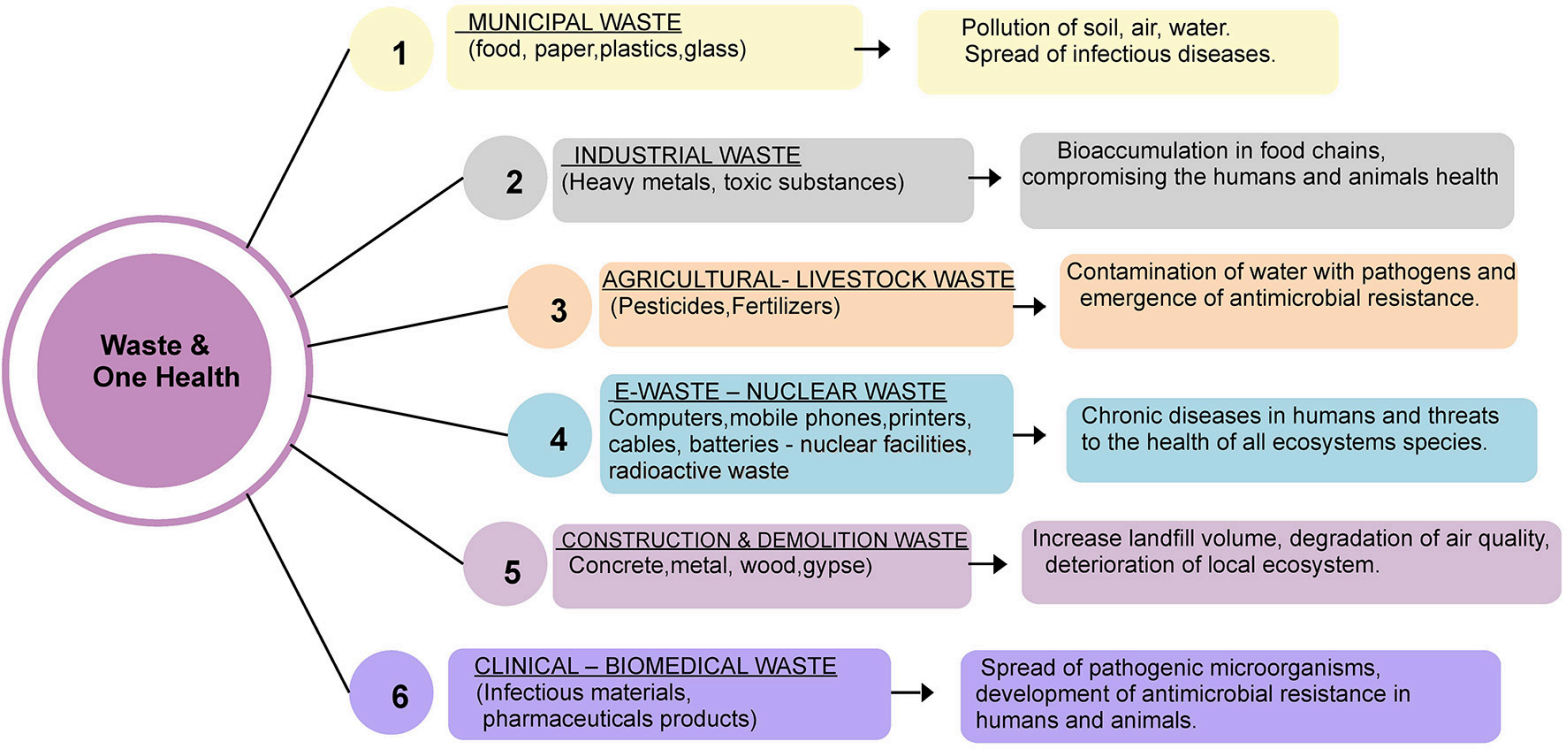
Waste management and one health approach.

The findings of this study highlight key patterns in waste management practices and their implications for human, animal, and environmental health. By examining different types of solid waste and their associated risks, the results provide evidence on how ineffective practices can contribute to pollution, disease transmission, and long-term ecological imbalance. At the same time, examples of community-based initiatives and policy measures are presented to illustrate potential pathways toward more effective and sustainable waste management aligned with the One Health approach (Figure 2).

### Industrial waste management

Industrial waste, arising from manufacturing, mining, textile, and chemical processes, may range from inert by-products to highly hazardous materials. Improper disposal contaminates ecosystems with heavy metals and toxic substances, which bioaccumulate in food chains and ultimately affect human and animal health, demonstrating the interconnectedness of environment, food safety, and wellbeing. Chemical pollutants, particularly heavy metals such as lead, mercury, cadmium, and arsenic, originate from a variety of anthropogenic sources. Industrial processes (e.g., mining, metal processing, chemical manufacturing), electronic waste, improper disposal of batteries and paints, and certain agricultural practices (use of fertilizers and pesticides) are major contributors. Once released into the environment, these metals can accumulate in soil, water, and biota, entering the food chain through crops, livestock, and aquatic organisms. Chronic exposure in humans can lead to neurological, renal, and cardiovascular disorders, while animals may experience reproductive and developmental effects, demonstrating the interconnected risks to human and animal health. Once released, heavy metals persist in the environment due to their non-biodegradable nature (7).

Despite a 90% reduction in average lead exposure among children in the United States since the 1970s, certain neighborhoods remain significantly affected, with children still experiencing harmful levels of lead exposure and animals also suffering from poisoning. These areas are characterized by a high concentration of commercial and industrial activities related to the use of lead, a history of heavy traffic, old and dilapidated buildings, and the presence of inactive or operational landfills, waste disposal sites, and hazardous waste facilities. Additionally, the quality of drinking water is often negatively affected by lead. The local population primarily belongs to socioeconomically vulnerable groups, lower-income and minority populations. Urban wildlife and domesticated animals are particularly vulnerable to lead contamination. In cities, lead accumulates in soil, buildings, dust, and even trees, and this issue will persist unless targeted actions are taken to eliminate these contamination sources (8).

Advancements in technology and economic growth have influenced the volume of post-production waste. Among the different categories, post-industrial waste—particularly from mining, metallurgy, and energy sectors—accounts for the largest proportion. Certain hazardous and non-hazardous waste materials can be repurposed for construction through “solidification/stabilization” processes, either as raw materials or as additives. However, the practice of integrating these waste materials into construction remains limited. Special attention should be given to fluoride-containing waste, as the reuse of solid fluoride waste has become a high priority. Fluoride is one of the few trace elements that has attracted considerable attention due to its harmful effects on the environment and on the health of humans and animals. Industrial discharge of effluents containing F– ions into surface waters further increases fluoride concentrations and contributes to environmental pollution. Therefore, the development of efficient and robust technologies for the removal of excess fluoride from aquatic environments is of critical importance (9).

Health concerns related to industrially contaminated sites (ICS) remain a significant public issue. Risk assessments typically focus on individual pollutants, although epidemiological studies provide substantial evidence of health risks among workers in industrial activities and local residents. Vulnerable groups—such as children, elderly women, and individuals with lifestyles that increase their exposure—may experience heightened risks despite not being directly employed in these industries. Waste disposal and treatment activities, along with industrial and commercial operations, constitute the two primary sources of soil contamination, jointly accounting for ~70% of identified polluted sites. Industrial and Commercial Sites (ICSs) encompass a wide range of facilities, including municipal and industrial landfills, large-scale industrial complexes (e.g., steelworks), petrochemical plants, waste incinerators, port areas, and active or decommissioned mining operations. Chemical contaminants most frequently detected in the vicinity of ICSs include heavy metals (e.g., arsenic, cadmium, lead, chromium), volatile organic compounds (VOCs, such as benzene and toluene), polycyclic aromatic hydrocarbons (PAHs, such as benzo[a]pyrene), dioxins, mineral oils, chlorinated hydrocarbons (CHCs, such as trichloroethylene and polychlorinated biphenyls–PCBs), and pesticides. According to a recent systematic review on the health impacts of hazardous waste, limitations in exposure assessment remain a major concern, affecting the reliability of health risk evaluations. Most epidemiological studies employ proximity-based indicators for exposure estimation, whereas more advanced approaches—such as atmospheric pollution dispersion modeling, soil quality monitoring, and human biomonitoring—have been implemented in only a small number of investigations. International recommendations emphasize that exposure assessment should consider all potential pathways and strive to integrate both direct methods (e.g., personal exposure monitoring) and indirect methods [e.g., microenvironmental monitoring and mathematical modeling; (7, 10)].

### Clinical and biomedical waste: challenges in safe disposal

Biological pollutants include pathogenic bacteria, viruses, parasites, and fungi that may be present in municipal, agricultural, and healthcare waste. Improper management of such wastes—through open dumping, untreated wastewater, or inadequate sterilization of biomedical waste—facilitates the spread of zoonotic pathogens and antimicrobial-resistant microorganisms. These pathogens can be transmitted directly to humans or animals or indirectly via contaminated food, water, and environmental surfaces, highlighting the cross-species and environmental pathways of disease transmission. Biomedical and healthcare waste, originating from hospitals, clinics, and laboratories, poses immediate risks when infectious materials, pharmaceuticals, and sharps are not properly managed. The unsafe disposal of such waste facilitates the spread of pathogens and contributes to the circulation of antimicrobial resistance genes, threatening the effectiveness of medical treatments for both humans and animals (11).

Clinical waste (CW) poses serious environmental and public health concerns. Proper waste management systems are essential for the safe disposal of hazardous medical waste. While incineration effectively eliminates pathogens and reduces waste volume, it produces clinical waste ash (CWA), a byproduct that increases environmental concentrations of heavy metals, inorganic salts, and organic compounds. The generation of CWIFA is expected to rise both nationally and globally. Uncontrolled disposal of these ashes causes significant damage because the toxicity of heavy metals and the presence of dioxins and furans contaminate soil as well as surface and groundwater. To address this issue, various studies have investigated the use of CW incinerator fly ash (CWIFA) in cement and concrete, with results indicating that CWIFA can be successfully incorporated into cement and concrete systems. Since the ashes have low chemical reactivity, further research is needed to enhance their activity by increasing their surface area or by using chemical activators to trigger pozzolanic reactions in cement-concrete systems. CWIFA has also shown promise as a fertilizer—because the ash contains macro- and micronutrients in addition to carbon and nitrogen—and as an inert material for road construction and asphalt. Therefore, more research is required to identify additional sustainable options for the disposal and utilization of ashes generated by CW incinerators. Leaching tests demonstrate that heavy metals are stabilized and immobilized in various cement-based systems. Moreover, detailed studies on heavy metal leachability and management strategies for metal residues in the leachate should be conducted to ensure proper handling and safe use of CW ashes, protecting both health and the environment. To address this issue, further research is needed to assess the impact of ash leachate, improve disposal methods, and explore innovative ways to recycle and repurpose ash in construction and other industries (12).

Biomedical waste, which encompasses industrial, hospital, and healthcare facility waste, presents a heightened risk of contamination and injury compared to other waste types. Promoting a culture of responsibility and sustainability can contribute to safer waste management practices, ultimately protecting both the environment and future generations. Healthcare facilities generate a wide array of waste materials, each presenting varying degrees of risk. Proper waste segregation is essential to classify waste into distinct streams, including infectious, hazardous, and general waste. Hazardous waste—such as sharps and infectious materials—must be handled exclusively by trained personnel using appropriate personal protective equipment (PPE). Effective biomedical waste management (BMWM) significantly reduces the risk of occupational exposure and mitigates the spread of infections among waste handlers and healthcare workers. A critical component of BMWM is the treatment and disposal of waste to ensure that the materials generated within healthcare institutions pose no threat to human health or the environment. This process is regulated by Biomedical Waste Management Rules, which provide guidelines for safe and environmentally responsible handling practices. Under controlled high-temperature incineration, biomedical waste undergoes thermal destruction, effectively reducing microbial load and substantially decreasing waste volume (13).

Biological pollutants illustrate how environmental, animal, and human health are tightly linked. Zoonotic pathogens from agricultural or healthcare waste can infect humans and domestic animals, while antimicrobial-resistant bacteria can circulate through soil, water, and food chains. Addressing these challenges requires coordinated strategies that combine safe waste handling, vector control, surveillance, and antimicrobial stewardship, aligning directly with the One Health approach.

### e-waste and nuclear waste

Electronic waste (e-waste) encompasses a wide variety of products that have reached the end of their useful life, including household appliances such as televisions, refrigerators, and washing machines, personal electronic devices such as computers, mobile phones, and printers, medical equipment, and electronic components like batteries, cables, and circuit boards. Improper disposal or recycling of e-waste can release hazardous substances, including heavy metals such as lead, mercury, cadmium, and arsenic, chemical compounds such as polychlorinated biphenyls (PCBs), polybrominated diphenyl ethers (PBDEs), and per- and polyfluoroalkyl substances (PFAS), and, in some cases, radioactive materials from older devices. Unsafe recycling practices, such as open-air burning or acid leaching to recover metals, can severely contaminate soil, water, and air, posing significant risks to human and animal health and disrupting ecosystems. Hazardous substances released from e-waste, including heavy metals and persistent organic pollutants, can accumulate in soils, water, and the food chain, affecting crops, livestock, and aquatic organisms. Humans consuming contaminated food or exposed to polluted environments may experience neurological, renal, and cardiovascular effects, while wildlife and domestic animals may suffer reproductive, developmental, and behavioral impacts (14).

The number of people exposed to hazardous substances due to unsafe and improper e-waste management practices continues to rise. This exposure has been linked to various health issues, including thyroid dysfunction, cellular damage, adverse neonatal outcomes, behavioral changes, and impaired lung function. Hazardous constituents present in electronic waste exert profound neurodevelopmental and neurobehavioral impacts, particularly in pediatric populations. Compounds such as polybrominated diphenyl ethers (PBDEs), polychlorinated biphenyls (PCBs), polycyclic aromatic hydrocarbons (PAHs), lead, cadmium, and mercury have been implicated in reduced intelligence quotient (IQ) scores and diminished cognitive performance. Furthermore, PBDEs, PCBs, mercury, and cadmium have been associated with neurodevelopmental abnormalities. Childhood exposure to PCBs, lead, mercury, and aluminum has also been linked to adverse mental health outcomes, including behavioral disorders, attention deficits, hyperactivity, and conduct problems. Studies indicate an increased prevalence of spontaneous abortions, stillbirths, premature births, and lower birth weights associated with e-waste exposure. Additionally, individuals living in or working within e-waste recycling areas show signs of significant DNA damage (15).

Radioactive waste and thermal discharges from nuclear facilities can persist in the environment for decades or centuries, creating reservoirs of contamination that affect ecosystems, animal populations, and human communities. Nuclear facilities, including power reactors, produce electricity through nuclear fission and, while they do not directly emit conventional air pollutants, they have important environmental impacts. Radioactive waste generated by these facilities remains hazardous for thousands of years and requires secure long-term management. Thermal discharges into water bodies can cause local ecosystem disruption, and the overall lifecycle of nuclear power, including uranium mining, fuel processing, facility construction, and decommissioning, contributes to environmental pressures (16).

Globally, activities related to the nuclear fuel cycle—including the operation and decommissioning of nuclear facilities—generate high-level radioactive waste, posing severe risks to human health and the environment. Exposure to radiation from radioactive waste can have harmful health effects due to ionizing radiation, such as increased cancer risk, chromosomal deletions in humans, and potential genetic defects in children. It can interfere with the repair of DNA, mRNA, and proteins, and may cause damage to the thyroid gland. Its tendency for long biological half-lives and high relative biological effectiveness makes it particularly damaging to tissues. One widely adopted method for managing this waste is cementation, which facilitates its encapsulation, solidification, and eventual disposal. The safe and well-organized management of radioactive waste is of utmost importance, so greater attention must be given to developing protective barriers. Cementitious binders for immobilizing radioactive waste offer a solution that is both stable and cost-effective. Potassium–magnesium phosphate cements appear suitable for immobilizing radioactive concrete waste generated during the decommissioning of nuclear power plants. Magnesium phosphate cement is highly effective in rapidly solidifying higher-content and high-level liquid wastes, as well as radioactive substances, during nuclear emergency situations. However, further large-scale research and refinement are required to enhance its effectiveness and integration (17).

Addressing these risks requires integrated waste management strategies that not only improve technical disposal and recycling practices but also incorporate surveillance, monitoring, and policy measures across sectors. By considering the One Health perspective, interventions can simultaneously mitigate risks to human health, protect animal welfare, and preserve ecosystem integrity, ensuring that environmental contaminants do not compromise the health of interconnected populations.

### Polycyclic aromatic hydrocarbons (PAHs)-cement production and use

Polycyclic aromatic hydrocarbons (PAHs) are primarily generated through incomplete combustion of organic matter, including fossil fuels, biomass, tobacco, and waste materials. Major sources of PAHs in the environment include industrial emissions, vehicle exhaust, open burning of municipal and agricultural waste, and certain household activities such as cooking with solid fuels. PAHs are persistent and bioaccumulative, capable of adsorbing to soil particles, sediment, and particulate matter in air. Human exposure occurs mainly through inhalation, ingestion of contaminated food (particularly smoked or grilled items), and dermal contact. Chronic exposure is associated with carcinogenicity, mutagenicity, and endocrine disruption. Similarly, wildlife and domestic animals can accumulate PAHs through contaminated water, soil, and food, resulting in reproductive, developmental, and immunological effects (18).

Polycyclic aromatic hydrocarbons (PAHs) were among the first substances identified as carcinogenic and remain a primary concern at hazardous waste sites. Given their frequent detection, it is crucial to establish feasible and effective remediation strategies to mitigate their impact. Polycyclic aromatic hydrocarbons (PAHs) are a class of persistent organic pollutants composed of multiple fused aromatic rings, typically generated during the incomplete combustion of organic matter such as coal, petroleum, wood, and other fossil fuels. They were among the first environmental contaminants to be recognized as carcinogenic, with several congeners, including benzo[a]pyrene, classified as Group 1 carcinogens by the International Agency for Research on Cancer (IARC). Due to their hydrophobic nature and low biodegradability, PAHs tend to adsorb strongly to soil particles and sediments, leading to long-term persistence in terrestrial and aquatic environments. These properties, combined with their mutagenic and teratogenic potential, make them a primary concern at hazardous waste sites. Given their frequent detection in contaminated soils and groundwater, it is essential to develop and implement remediation strategies that are both technically feasible and cost-effective. Such strategies may include bioremediation approaches (e.g., microbial degradation), thermal desorption, chemical oxidation, and integrated treatment technologies to effectively reduce their environmental and human health risks. Addressing PAH contamination requires interdisciplinary approaches that simultaneously protect people, animals, and environmental integrity (19, 20).

Agricultural and livestock waste represents another critical category within the One Health framework. It includes crop residues, animal manure, fertilizers, and pesticide containers. Poor handling can contaminate water sources with pathogens, nitrates, and chemicals, drive greenhouse gas emissions, and accelerate the emergence of antimicrobial resistance. These consequences highlight the direct overlap of agricultural practices with human food safety, animal health, and environmental sustainability (4).

Construction and demolition (C&D) waste, composed of concrete, metals, wood, and gypsum, if not recycled or reused, increases landfill volumes and contributes to environmental degradation. While often considered less hazardous, its mismanagement still affects land use, air quality, and local ecosystems, indirectly influencing public health and the quality of shared environments. Cement production is a major contributor to greenhouse gas emissions worldwide, accounting for a substantial share of global CO_2_ emissions. This has heightened the urgency to develop alternative sustainable cementitious materials to reduce the construction industry's environmental footprint. Geopolymer production does not involve clinker calcination or high-temperature kiln firing, rendering the process considerably more environmentally friendly. Furthermore, geopolymer technology enables the utilization of industrial by-products such as fly ash and ground granulated blast furnace slag (GGBFS). Consequently, the environmental assessment of geopolymers has gained increasing attention over the past decades, with Life Cycle Assessment (LCA) being the most widely adopted methodology for systematically evaluating environmental impacts from raw material extraction through production, use, and end-of-life disposal. Current findings indicate that the composition of the alkaline activator and the source of fly ash are critical parameters that warrant careful consideration to further enhance the sustainability of geopolymer mixtures. To facilitate the transition toward a more sustainable, energy-efficient, and comfortable built environment, in line with circular economy principles within the construction sector, further research and optimization of materials are necessary, particularly for building envelope applications. Future studies should focus on identifying greener alternatives to sodium silicate as an alkaline activator—for example, by employing renewable energy sources in its energy-intensive production—and developing more efficient recovery methods for by-products such as fly ash, cenospheres, and GGBFS from their primary production processes (21, 22).

### One health and community engagement

In many regions, particularly in the global South, waste management practices often involve mixing domestic and commercial waste with hazardous materials during storage and handling. Additionally, waste is frequently stored in outdated or poorly maintained facilities. Inefficient solid waste management is closely associated with adverse public health outcomes and represents a critical constraint to environmental quality and the sustainable growth of urban areas. Optimizing community engagement in integrated solid waste management necessitates fostering favorable public perceptions and attitudes. Civil society actors, including non-governmental and community-based organizations, can play a pivotal role in advancing waste minimization strategies, promoting source segregation and material sorting, and facilitating the reuse and recycling of resources. Also, raising awareness through print, digital, and social media campaigns is essential to encourage individuals to adopt proper waste disposal practices (23).

Government-funded environmental protection initiatives may gradually be overtaken by community-driven programs. The recurring environmental and economic benefits of such grassroots efforts can serve as a foundation for fostering long-term waste reduction and sustainability practices at the local level. The findings support that sustainable, community-based intervention programs possess significant potential to overcome the initial reservations of small and medium-sized enterprises regarding the adoption of source reduction measures, by demonstrating tangible benefits and gradually building trust. Additionally, through the enhancement and promotion of modern community health aimed at preventing chronic diseases, by writing and distributing educational material, creating specialized programs in mass media, organizing seminars with various groups and social organizations, and focusing on the principles of prevention, recycling, and reuse, we can address the adverse consequences in a holistic and coordinated manner, gradually and effectively.

Collaboration with community groups in implementing these programs leads to overall benefits for the community and encourages participation from small businesses, although achieving widespread acceptance requires time and sustained effort. Studies serve as a foundation for broader future initiatives, potentially on a global scale. Moreover, community-based programs can represent a valuable alternative to conventional government-funded environmental initiatives, steadily advancing local sustainability through recurring environmental and economic benefits (24, 25).

## Discussion-conclusions

Effective community-based waste management has been successfully demonstrated through a variety of practices, including source separation programs in schools, public spaces, and businesses, as well as the promotion of community composting initiatives for organic waste. Circular economy models that emphasize material reuse and product repair further reduce the demand for raw resources while minimizing waste generation. Additional strategies such as the establishment of specialized collection centers for electronic devices and hazardous waste, combined with educational campaigns that raise awareness on prevention and reduction, have proven essential in engaging local populations and improving sustainability outcomes (26).

From a policy and practice perspective, several measures are required to address the complex challenges of waste management. To reduce the volume of waste ending up in landfills, recycling and composting initiatives must be strengthened and scaled. To prevent environmental contamination from hazardous and electronic waste, safe collection and disposal systems should be legally enforced and properly monitored. To limit the spread of pathogens and the development of antimicrobial resistance, strict protocols for disinfection and safe handling of biomedical waste are indispensable (29, 30). Furthermore, to enhance environmental justice, policies should guarantee equitable access to clean and safe waste management infrastructure across all communities. Equally important are incentives that encourage citizen participation, such as subsidies, tax benefits, and deposit-return schemes, which foster behavioral change at the community level. Finally, to improve the monitoring of environmental risks, the integration of digital technologies and telemonitoring systems (e-Health and e-Environment) is recommended, as these approaches allow for real-time data collection, early detection of hazards, and more effective decision-making (27).

Despite the growing body of literature on solid waste management and its implications for public health and the environment, several important gaps remain that urgently need to be filled such as: First, there is a lack of integrated research linking waste management practices directly to the One Health framework, especially studies that simultaneously evaluate human, animal, and environmental health outcomes. Second, while the health effects of specific pollutants such as heavy metals, PAHs, and biomedical waste have been documented, there is limited evidence on their synergistic and long-term impacts across ecosystems and food chains. Third, the field lacks comprehensive data from low- and middle-income countries, where informal waste management practices and inadequate infrastructure exacerbate risks but are underreported in the literature. Furthermore, the role of emerging waste streams such as electronic waste and radioactive residues in driving antimicrobial resistance or chronic disease pathways is not yet sufficiently understood (31). Another critical gap is the evaluation of community-based interventions and policy tools (e.g., deposit-return schemes, digital monitoring systems) in terms of their measurable health and environmental benefits. Finally, there is limited exploration of interdisciplinary approaches and digital innovations, such as e-Health and e-Environment platforms, that could provide real-time monitoring, early detection of hazards, and improved governance (28, 32).

In conclusion, minimizing hazardous waste according to One Health Approach provides multiple important benefits. It allows for the incorporation of environmental health and safety considerations within an integrated framework for risk assessment. This approach facilitates the evaluation of risks during both normal operations and emergency situations, enabling the identification of the most urgent threats that require prompt corrective action. Furthermore, it supports the gradual implementation of a comprehensive risk management strategy in sectors facing environmental and safety challenges, such as waste collection. At the same time, it encourages the development of new skills and innovative approaches aimed at the gradual implementation of an integrated risk management approach in areas facing immediate but also long-term environmental challenges.
